# Disturbing effect of cepharanthine on valve interstitial cells calcification *via* regulating glycolytic metabolism pathways

**DOI:** 10.3389/fphar.2022.1070922

**Published:** 2022-11-17

**Authors:** Fei Xie, Juanjuan Han, Dashuai Wang, Peng Liu, Chao Liu, Fuqiang Sun, Kang Xu

**Affiliations:** ^1^ Department of Cardiovascular Surgery, The First Affiliated Hospital of Zhengzhou University, Zhengzhou, China; ^2^ Hubei Engineering Technology Research Center of Chinese Materia Medica Processing, College of Pharmacy, Hubei University of Chinese Medicine, Wuhan, China; ^3^ Department of Cardiovascular Surgery, Fuwai Central China Cardiovascular Hospital, Henan Provincial People’s Hospital, Henan Cardiovascular Hospital of Zhengzhou University, Zhengzhou, China

**Keywords:** calcific aortic valve disease, osteogenesis, glycolysis, metabolism, natural product

## Abstract

Osteogenic differentiation of valve interstitial cells (VICs) directly leads to aortic valve calcification, which is a common cardiovascular disease caused by inflammation and metabolic disorder. There is still no ideal drug for its treatment and prevention. The purpose of this study was to explore the effect and molecular mechanism of cepharanthine (CEP), a natural product, on inhibiting the osteogenic differentiation of VICs. First, CCK8 assay was used to evaluate cell viability of CEP on VICs. CEP concentration of 10 μM was the effective dose with slight cytotoxicity, which was used for further study. The alizarin red staining analysis showed that CEP significantly inhibited calcium deposition caused by osteogenic medium related calcification induction. In order to explore the anti-calcification molecular mechanism of CEP, transcriptome and metabolome were synchronously used to discover the possible molecular mechanism and target of CEP. The results showed that CEP inhibited valve calcification by regulating the glycolytic pathway. The molecular docking of CEP and selected key factors in glycolysis showed significant binding energies for GLUT1 (−11.3 kcal/mol), ENO1 (−10.6 kcal/mol), PKM (−9.8 kcal/mol), HK2 (−9.2 kcal/mol), PFKM (−9.0 kcal/mol), and PFKP (−8.9 kcal/mol). The correlation analysis of RUNX2 expression and cellular lactate content showed R^2^ of 0.7 (*p* < 0.001). In conclusion, this study demonstrated that CEP inhibited osteoblastic differentiation of VICs by interfering with glycolytic metabolisms *via* downregulation of the production of lactate and glycolysis-associated metabolites.

## Introduction

Calcific aortic valve disease (CAVD) is a common heart valve disorder, characterized by calcium deposition, osteogenic lesion, and fibrocalcific changes in the valve leaflet. CAVD is a gradually progressive condition, ranging from chronic inflammation to osteogenic lesions and then leaflet calcification and thickening, culminating in aortic stenosis, heart failure, and ultimately premature death. CAVD is the third leading cause of heart disease, affecting nearly 1.5 million people and resulting in 100,000 heart valve replacement surgeries in the U.S. alone. In recent years, the incidence of CAVD has gradually increased, and its epidemic area has gradually increased. The prevention and treatment of CAVD are of great significance. Currently, surgical intervention is the only effective treatment for CAVD in the late stage, but there is no effective intervention for early CAVD treatment. Although a few drugs have been considered for CAVD, such as statins and angiotensin-converting enzyme inhibitors, a number of large-scale clinical trials have confirmed that neither of them can effectively prevent the progression of the disease. Thus, new drugs are needed to block the progression of CAVD.

Although CAVD is a complex and multifactorial disease, there is much evidence suggesting that inflammatory reaction is the key initiating factor of valve calcification (Cho et al., 2018). CAVD is an active disease process driven by valve interstitial cells (VICs) in the valve tissue under the inflammatory state (Xu et al., 2018; Liu et al., 2020). It has been suggested that chronic inflammation significantly promotes the expression of osteogenic marker genes (such as BMP-2 and RUNX2) in VICs, thereby leading to the deposition of valve matrix apatite, finally leading to calcification of the valve tissue (Deng et al., 2016). Therefore, inhibition of inflammatory response may delay the progression of valve disease. Clinical research has shown that traditional anti-inflammatory drugs provide only limited curative effects, and drug resistance easily develops in most of the chronic inflammatory diseases (Rane et al., 2019), such as osteoarthritis (Puljak et al., 2017), rheumatoid arthritis (Nurmohamed and Dijkmans, 2005), and ulcerative colitis (Klein and Eliakim, 2010). Therefore, there is an urgent need for effective and safe medications from natural compounds. There is growing evidence that multiple active ingredients in Chinese herbal medicine can be beneficial for preventing CAVD.

Cepharanthine (CEP) is a natural small molecule alkaloid extracted from the plant *Stephania japonica* (Thunb.) Miers. It has unique antioxidant, antiviral, and immunomodulatory pharmacological properties in multiple diseases (Bailly, 2019). CEP can protect nuclear DNA from the damages induced by endogenous oxidants *via* its antioxidant properties (Halicka et al., 2008). Okamoto et al. found that CEP is a highly potent inhibitor of HIV-1 replication in a chronically infected monocytic cell line (Okamoto et al., 1998). What’s more, CEP was recently confirmed to be a potential natural antiviral compound for the prevention and treatment of SARS-CoV-2 and SARS-CoV infection (Ruan et al., 2021). CEP also exhibits significant anti-inflammatory activity (Kudo et al., 2011). Recent studies have indicated that CEP can inhibit the activation of NLRP3 inflammasome and nuclear factor-kappa B (NF-κB), nitric oxide production, and cyclooxygenase production, all of which are crucial to inflammatory response (Samra et al., 2016). Lin et al. reported that CEP could inhibit the expression of osteoclast-differentiation marker genes in osteoclasts (Lin et al., 2018). Therefore, this study aimed to investigate whether CEP can reverse the osteogenic lesions of VICs induced by osteogenic medium (OM) and determine the underlying mechanism.

In this study, we developed an *in vitro* CAVD model of VICs (with OM induction) to assess the inhibitory effects of CEP on calcification caused by abnormal gene/protein phenotypic changes. To further unravel the underlying molecular mechanisms, we detected the gene expression profiles and metabolites of VICs after CEP treatment. According to transcriptomic and metabonomic results, CEP downregulated the genes and metabolites through the glycolytic metabolism pathway. This study provides a new approach for preventing valve calcification from the perspective of regulating glycolytic metabolism.

## Material and methods

### Cell culture

Aortic VICs were isolated and cultured as described in a previous study (Wang et al., 2021a). Briefly, heart aortic valve tissues were cut into small pieces and digested with 2 mg/ml type I collagenase (Sigma-Aldrich, St. Louis, MO, United States) for 3–4 h. After digestion, the cell suspension was filtered through a 70-µm filter (pluriSelect Life Science, El Cajon, CA, United States), and the cells were seeded in high-glucose Dulbecco’s modified Eagle medium (DMEM) supplemented with 10% fetal bovine serum (FBS, Gibco Laboratories, Gaithersburg, MD, United States). The thin and normal aortic valve tissues were collected from eight patients undergoing Bentall surgery to treat DeBakey I acute aortic dissection. All the eight issue samples were used for cell isolation. Cells from the second or third passage were utilized in cellular experiments.

### *In vitro* calcification model construction and cepharanthine treatment

Cepharanthine (CEP) was purchased from Selleck Chemicals (S4238, Selleck Chemicals, United States) and then dissolved in DMSO as a 10 mM stock solution. VICs were starved for 12 h with 2% FBS + DMEM and then incubated with different concentrations of CEP (1–100 μM) for 48 h; DMSO was used as control. Meanwhile, the cells were exposed to 10 μM CEP for 5 days to test the long-term cell cytotoxicity. In addition, VICs were cultured with OM to induce cell calcification (Zhou et al., 2020). VICs were seeded on six-well plates at a density of 10 × 10^4^ cells/well in DMEM/F12 supplemented with 10% FBS. After cell starvation, the cells were treated with OM medium with or without 10 μM CEP for 48 h; DMSO was used as control. In addition, OM was cultured with VICs to induce cell calcification. OM alone (calcification-induction group) or OM plus CEP at 10 μM (CEP-treated group) was used for treating VICs; DMSO was used as control. The cells sample was used for metabolome and transcriptome analysis, and further studies.

### Western blotting

The protein sample was extracted by RIPA protein extraction reagent (Beyotime, Beijing, China), and the protein concentration was determined by the enhanced BCA protein detection kit (Beyotime, Beijing, China). Equal amounts of protein were separated by SDS–PAGE gel electrophoresis and then transferred to PVDF membranes (0.2 μm; Bio-Rad) overnight. After blocking with 5% skimmed milk for 1 h, the membranes were incubated with primary antibody RUNX2 (CST, 8486s) at 4°C overnight. The membranes were incubated with secondary antibodies at room temperature for 1 h after washing three times with 1×TBST. Finally, target protein was visualized by ECL Western Detection Kit (Thermo Fisher Scientific, United States) and quantified by ImageJ software.

### RNA sequencing

RNA sequencing was performed on VICs subjected to different treatments to determine the changes in their mRNA expression profiles (Wang et al., 2022). The total RNA was extracted by RNA extraction kit (Thermo Fisher Scientific, Waltham, MA), and then sent to the BGI Co., Ltd. Shenzhen, China, where RNA-sequencing operation was performed on BGISEQ platform. All analyses were repeated for three times. The R language was used to further analyze the sequencing results to find differential genes in the chip expression profile. Then, we use the R program to perform gene ontology (GO) biological function enrichment analysis and genome encyclopedia (KEGG) signal pathway enrichment analysis.

### Gas chromatography–mass spectrometry metabolome analysis

Gas chromatography–mass spectrometry (GC-MS) was used to analyze the differential metabolites in VICs, as described in previous studies (Fiehn, 2016; Qu et al., 2022). In short, the samples were collected with −80°C precooled methanol solution (10 μL/ml) on ice and then frozen and thawed with liquid nitrogen for three times. After centrifugation, the supernatant was transferred to a new EP tube and dried with a nitrogen blower at 35°C. The dry samples were re-dissolved in 80 μL methoxy-pyridine solution (20 mg/ml) and 50 µL BSTFA. After derivation, the samples were incubated in a water bath at 80°C for 1 h and then transferred to 200 μL micro-inserts. Trace1300 GC-MS System (Thermo, United States) was employed for the metabolomic analysis, along with SIMCA software (Umetrics, Sweden). The control group was treated with DMSO, and eight samples were collected from each group.

### Molecular target analysis

All key proteases of metabolic pathways enriched by differential metabolites were examined as potential molecular targets of CEP. Glycolysis-associated targets were selected based on the KEGG database. The selected targets were tested using molecular docking with CEP *via* AutoDock Vina 1.2.2 in line with the previous study (Wang et al., 2020). The binding energy was listed for further analysis.

### Statistical analysis

All data were analyzed and expressed as the mean ± standard deviation (SD). Statistical comparisons were made by analysis of variance to evaluate differences among groups. A *p*-value lower than 0.05 was considered statistically significant.

## Results

### Cepharanthine inhibits valve interstitial cells calcification

**FIGURE 1:**
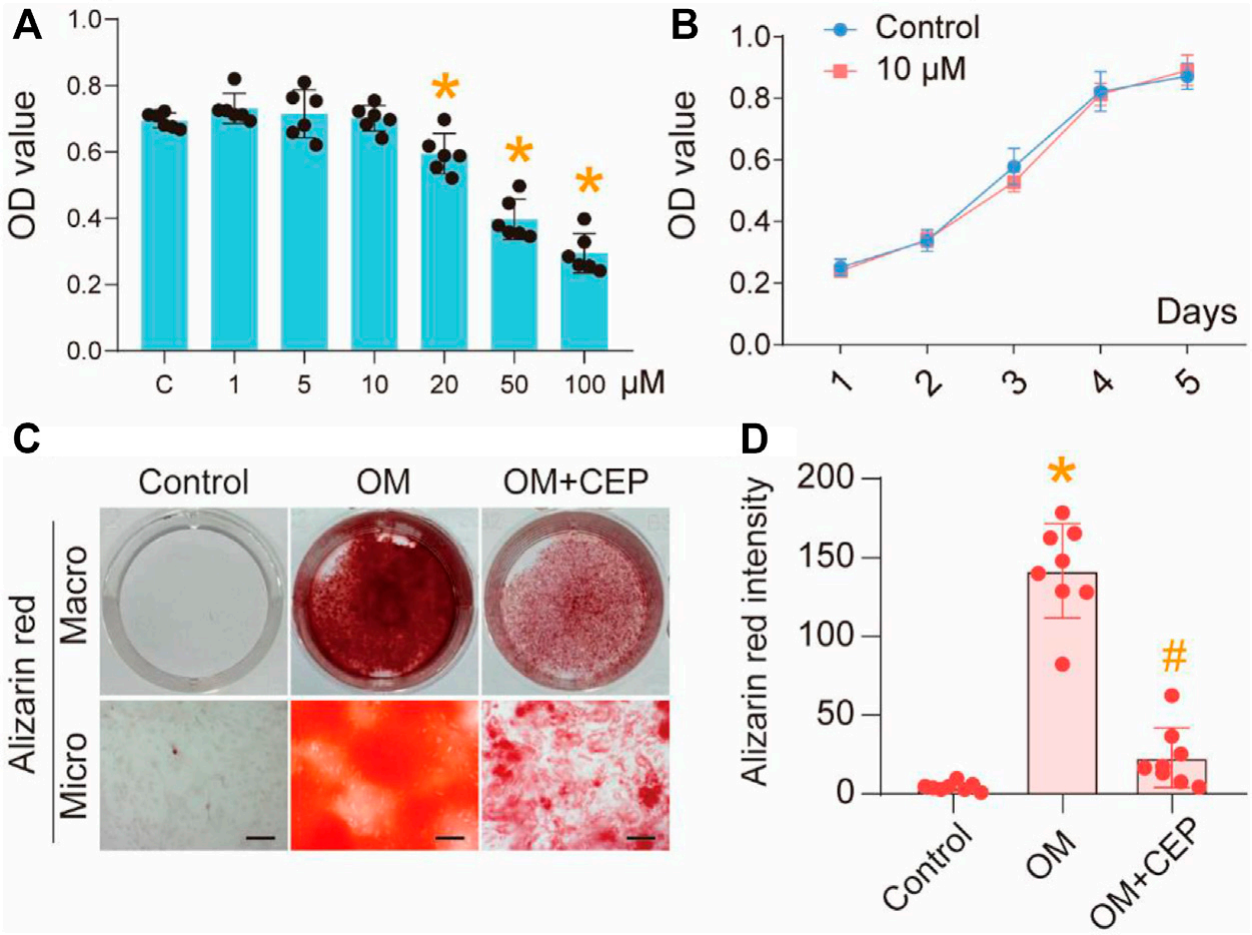
CEP inhibits osteogenic differentiation of VICs. **(A)** CCK8 assay of VICs with 1–100 μM CEP treatments, (*) *p* < 0.05 vs. C (control); **(B)** Cell proliferation testing with 10 μM CEP treatment; **(C)** Alizarin red staining of OM-induced VICs with or without CEP treatment, scale bar: 50 μm; **(D)** The intensity of alizarin red, statistical analysis. (*) *p* <0.05 vs. control, (#) *p* < 0.05 vs. OM indicate significant difference.

VICs were exposed to various concentrations of CEP (from 1 to 100 μM) to examine the effects of CEP on cell viability. We found that 20 μM CEP began to significantly inhibit the growth of cells relative to the control group (*P<0.05), while 50 and 100 μM CEP had obvious inhibitory effects (*p <0.05) (Figure 1A). Therefore, we selected 10 μM CEP for further studies. According to the cell growth curve of 1–5 days when treated with 10 μM CEP, compared with control, CEP did not affect the cell proliferation (Figure 1B). This indicated that 10 μM CEP showed no obvious cytotoxicity. In the OM-induced condition, after treating the VIC cells with CEP for 21 days, the alizarin red staining results showed that CEP significantly inhibited the osteoblastic differentiation of VICs compared with the OM-induced group (*p <0.05) (Figures 1C,D). These results suggest that CEP can inhibit the osteogenic differentiation of VICs.

### Gene expression profiles of valve interstitial cells under osteogenic medium plus cepharanthine treatment

**FIGURE 2:**
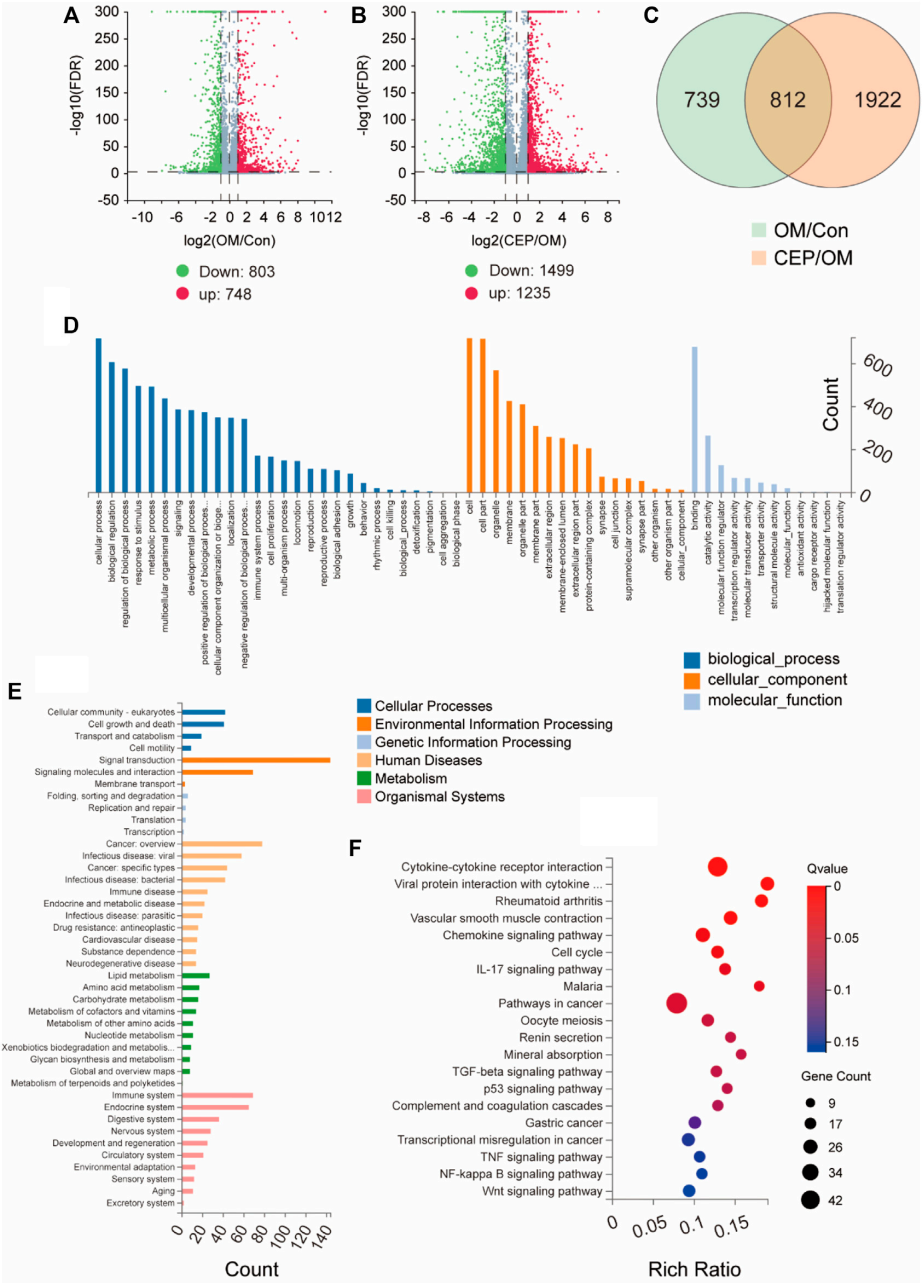
Gene expression analysis of VICs with or without CEP treatment under the OM. **(A)** and **(B)** Volcano plots based on gene expression of VICs with CEP treatments under the OM induction. Log2FC > 1; FDR < 0.001, red plot indicates upregulation, and green plot indicates downregulation; **(C)** Venn of differentially expressed genes (DEGs) indicate 812 common DEGs involved in the anti-calcification effect of CEP; **(D)** GO enrichment based on 812 common DEGs; **(E)** classification of enrichment; **(F)** KEGG pathway enrichment based on 812 common DEGs.

In order to explore the molecular mechanism by which CEP inhibits the calcification of VICs, we used RNA sequencing to analyze OM-induced VICs with or without CEP treatment. The analysis of differentially expressed genes (DEGs) showed that 748 genes were upregulated, and 803 genes were downregulated in VICs induced by OM. Compared with the OM group, 1,235 genes were upregulated and 1,499 genes were downregulated in CEP-treated VICs (Figures 2A,B). After Venn analysis, 812 common DEGs were identified (Figure 2C). The results of GO and KEGG enrichment analysis showed that the genes involved in anti-calcification effects of CEP were mostly enriched in the cytokine–cytokine receptor interaction, chemokine signaling pathway, and metabolism pathways (Figures 2D–F).

### Metabolomic profile of valve interstitial cells with osteogenic medium plus cepharanthine treatment

**FIGURE 3:**
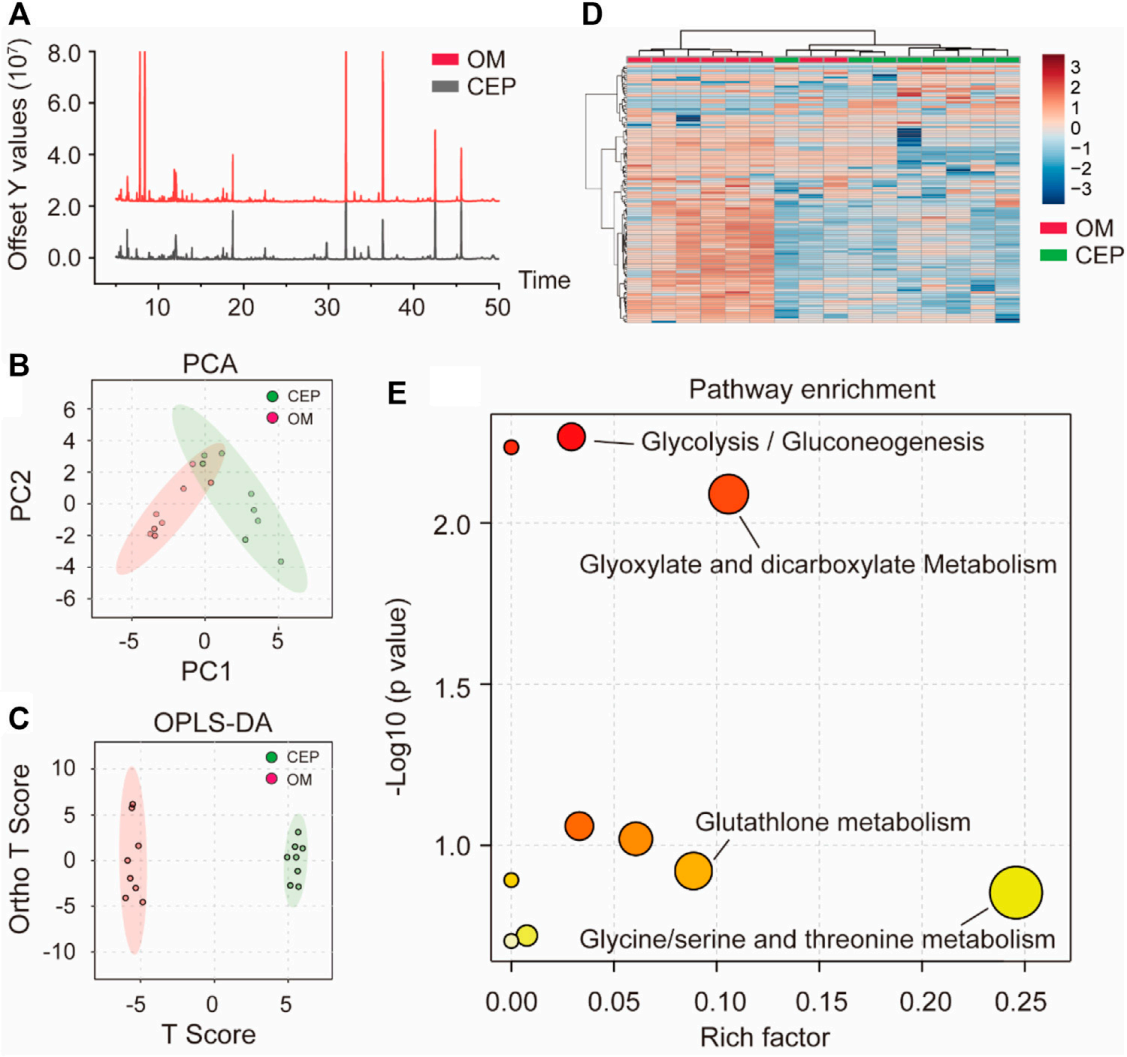
Metabolomic analysis of VICs with or without CEP treatment under the OM induction. **(A)** Total ion flow diagram; **(B)** PCA classification; **(C)** OPLS-DA classification; **(D)** the heat map analysis; **(E)** pathway enrichment analysis based on differential metabolites (VIP >1).

Based on GC–MS metabolome analysis, the total ion flow diagram showed the difference between the OM group and the CEP group (OM plus CEP treatment) (Figure 3A). Principal component analysis (PCA) and orthogonal partial least-squares discrimination analysis (OPLS-DA) macroscopically indicated the obvious differences in metabolites between the above two groups (Figures 3B,C). The heat map analysis showed two classifications based on OM and CEP groups (Figure 3D). Pathway enrichment analysis with differential metabolites (VIP >1) showed that glycolysis/gluconeogenesis, glyoxylate and dicarboxylate metabolism, glutathione metabolism, glycine/serine, and threonine metabolism were significantly enriched (Figure 3E).

### Cepharanthine interferes with glycolysis to prevent calcification

**FIGURE 4:**
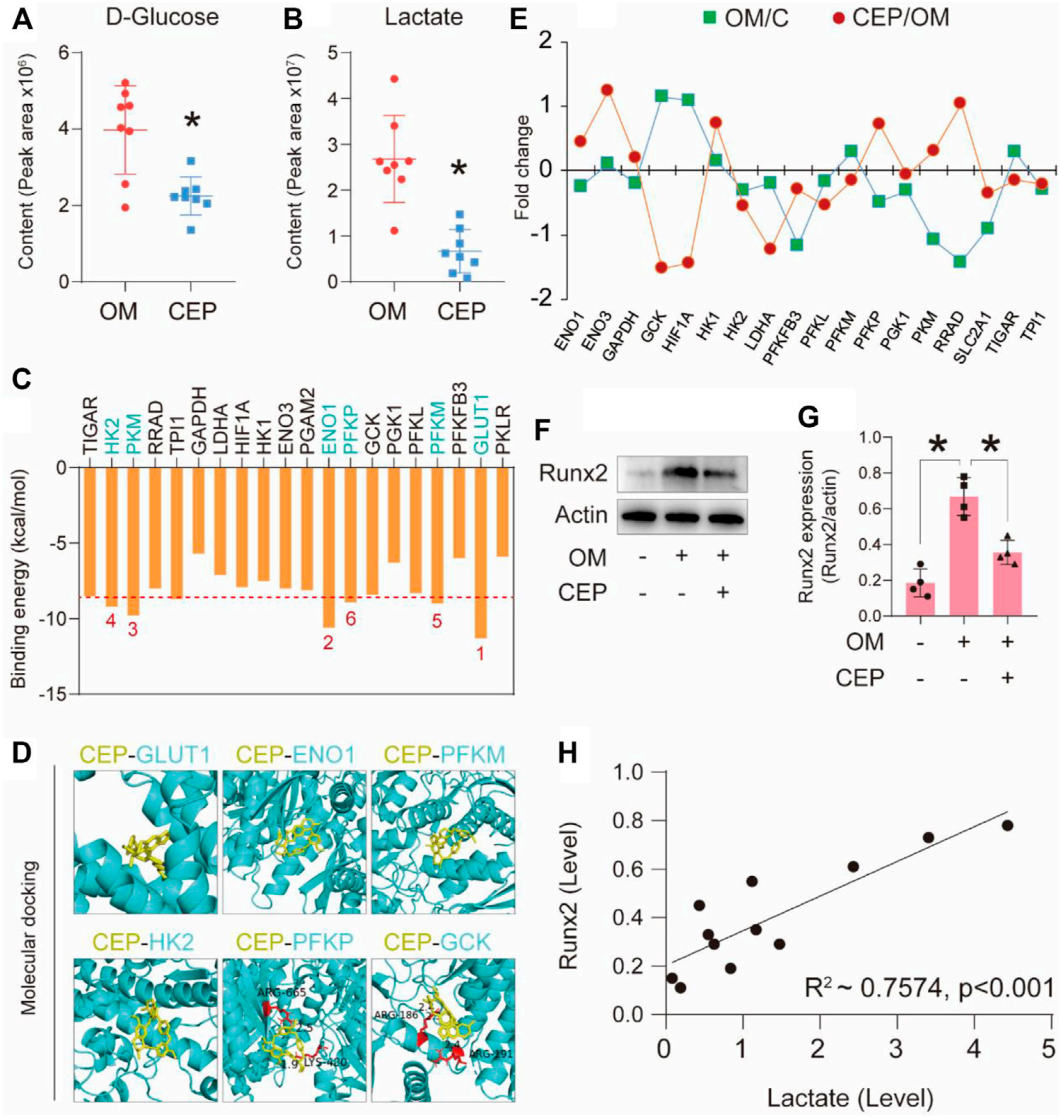
CEP interferes with the glycolysis pathway to confer anti-calcification effects. **(A)** and **(B)** The relative content of D-glucose and lactate based on the peak area in metabolomic analysis; (*) *p* < 0.05 indicates significant difference; **(C)** binding energy of each protein with CEP *via* molecular docking; **(D)** top six binding energy proteins and CEP docking details; **(E)** associated gene expression profiles of VICs treated with OM plus CEP in the glycolysis pathway; **(F)** and **(G)** Runx2 expression in VICs with CEP treatment under the condition of OM induction; (*) *p* < 0.05 indicates significant difference; **(H)** correlation analysis based on lactate level and Runx2 expression level.

Furthermore, we selected two differential metabolites of VICs associated with glycolysis and compared the peak areas of D-glucose and lactate. These two metabolites in CEP-treated samples had significantly lower peak areas than those in the OM samples (Figures 4A,B). The molecular docking of CEP and selected key factors in the glycolysis pathway showed significant binding energies for GLUT1 (−11.3 kcal/mol), ENO1 (−10.6 kcal/mol), PKM (−9.8 kcal/mol), HK2 (−9.2 kcal/mol), PFKM (−9.0 kcal/mol), and PFKP (−8.9 kcal/mol) (Figure 4C). The molecular details of these top six binding proteins are shown in Figure 4D. The gene expression levels of the selected factors were extracted from the above RNA-sequencing data, and showed that CEP significantly regulated the glycolysis-associated gene expression (Figure 4E). Western blotting assay of Runx2 (calcification marker) indicated that CEP obviously inhibited VICs calcification (*p* <0.05) (Figures 4F,G). The correlation analysis of Runx2 expression and lactate content showed R^2^ of 0.7 (*p* < 0.001) (Figure 4H). This finding indicated that the anti-calcification effect of CEP was linked with lactate production, that is, indirectly associated with glycolysis.

## Discussion

In this study, we found that cephalanthine may inhibit aortic valve calcification. Through the cell viability testing, we found that the concentration greater than 10 μM had obvious suppressive effect on the cellular activity of VICs. To avoid cytotoxicity, we chose 10 μM CEP as the final concentration in the follow-up mechanistic experiments. CEP at 10 μM concentration showed a significant inhibitory effect on osteoblastic differentiation of VICs according to the alizarin red staining.

In order to further investigate the anti-calcification mechanism of CEP, transcriptome and metabolome analysis were performed concurrently. We found that cephalanthine regulated the glycolysis pathway of VICs. Previous studies have shown that glycolysis plays an important role in many diseases (Ghazi et al., 2019; Abbaszadeh et al., 2020; Tang, 2020). It has been reported that the change of glycolysis can promote the progression of nonalcoholic fatty liver disease to nonalcoholic steatohepatitis, and finally to cirrhosis and hepatocellular carcinoma. For tumor growth, cancer cells metabolize glucose through glycolysis under aerobic conditions, and then rapidly generate ATP to obtain a lot of energy (Bose and Le, 2018). Other studies have shown that acute renal injury and diabetic nephropathy are characterized by mitochondrial dysfunction and accumulation of glycolytic intermediates, which can be metabolized as toxic end products (Qi et al., 2017; Scantlebery et al., 2021). Therefore, glycolysis plays an important regulatory role in various diseases. In cardiovascular diseases, because glycolysis is the most important energy metabolism mode of endothelial cells, previous studies have shown that the increase of aerobic glycolysis in endothelial cells in areas prone to vascular atherosclerosis is the cause of inflammation and atherosclerosis (Yang et al., 2018). So far, there has been no research report on the relationship between glycolysis and valve calcification.

Previous studies have shown that the activation of VICs for proliferation and differentiation, and significant promotion of the expression of key calcification genes (BMP-2 and RUNX2) in VICs, would lead to the deposition of valve matrix apatite, finally causing calcification of the valve tissue (Xu et al., 2020; Gonzalez Rodriguez et al., 2021). Therefore, VICs are the direct participants in valve calcification. According to our previous studies, inhibition of osteoblastic lesions of VICs can significantly inhibit calcium deposition in the valve tissue (Xu et al., 2019). In this study, we found that CEP was able to inhibit the osteogenic differentiation of VICs. We used transcriptome and metabolome analysis to further explore the mechanism of drug inhibition of valve calcification from the perspective of the relationship between inflammation and metabolism.

Through the exploration of the mechanism based on the multi-omics study, we found that OM induced calcification, which led to a large accumulation of glucose and lactate in VICs. CEP treatment significantly reduced the content of glucose and lactate. Correlation analysis showed that the decrease of lactate level significantly positively correlated with the expression of RUNX2 (calcification marker). In the process of valve calcification, the disorder of the glycolysis pathway in VICs resulted in the accumulation of a large number of lactate and other products, which may be an important reason for the process of valve calcification. Certo summarized the metabolic pathway regulated by high lactate level and explained that it was closely related to the development of chronic inflammatory diseases (Certo et al., 2021). According to our early studies, chronic inflammation is the direct cause of aortic valve calcification (Wang et al., 2021b). Therefore, it is reasonable to believe that inhibiting the lactate and associated metabolite production can inhibit valve calcification.

In conclusion, CEP can inhibit the lactate and associated metabolite production in VICs, and regulate the glycolytic pathway, thereby inhibiting the osteoblastic differentiation of VICs. CEP can be used as a potential drug to treat CAVD.

## Data Availability

The datasets presented in this study can be found in online repositories. The names of the repository/repositories and accession number(s) can be found below: https://www.ncbi.nlm.nih.gov/, PRJNA890260.
